# COX5A Alleviates Doxorubicin-Induced Cardiotoxicity by Suppressing Oxidative Stress, Mitochondrial Dysfunction and Cardiomyocyte Apoptosis

**DOI:** 10.3390/ijms241210400

**Published:** 2023-06-20

**Authors:** Peipei Zhang, Hao Lu, Yuan Wu, Danbo Lu, Chenguang Li, Xiangdong Yang, Zhangwei Chen, Juying Qian, Junbo Ge

**Affiliations:** Department of Cardiology, Zhongshan Hospital, Fudan University, Shanghai Institute of Cardiovascular Diseases, National Clinical Research Center for Interventional Medicine, Shanghai 200032, China

**Keywords:** COX5A, doxorubicin, oxidative stress, apoptosis, mitochondrial dysfunction

## Abstract

Doxorubicin (DOX) as a chemotherapeutic agent can cause mitochondrial dysfunction and heart failure. COX5A has been described as an important regulator of mitochondrial energy metabolism. We investigate the roles of COX5A in DOX-induced cardiomyopathy and explore the underlying mechanisms. C57BL/6J mice and H9c2 cardiomyoblasts were treated with DOX, and the COX5A expression was assessed. An adeno-associated virus serum type 9 (AAV9) and lenti-virus system were used to upregulate COX5A expression. Echocardiographic parameters, morphological and histological analyses, transmission electron microscope and immunofluorescence assays were used to assess cardiac and mitochondrial function. In a human study, we found that cardiac COX5A expression was dramatically decreased in patients with end-stage dilated cardiomyopathy (DCM) compared to the control group. COX5A was significantly downregulated following DOX stimulation in the heart of mice and H9c2 cells. Reduced cardiac function, decreased myocardium glucose uptake, mitochondrial morphology disturbance, reduced activity of mitochondrial cytochrome *c* oxidase (COX) and lowered ATP content were detected after DOX stimulation in mice, which could be significantly improved by overexpression of COX5A. Overexpression of COX5A effectively protected against DOX-induced oxidative stress, mitochondrial dysfunction and cardiomyocyte apoptosis in vivo and in vitro. Mechanistically, the phosphorylation of Akt (Thr308) and Akt (Ser473) were also decreased following DOX treatment, which could be reserved by the upregulation of COX5A. Furthermore, PI3K inhibitors abrogated the protection effects of COX5A against DOX-induced cardiotoxicity in H9c2 cells. Thus, we identified that PI3K/Akt signaling was responsible for the COX5A-mediated protective role in DOX-induced cardiomyopathy. These results demonstrated the protective effect of COX5A in mitochondrial dysfunction, oxidative stress, and cardiomyocyte apoptosis, providing a potential therapeutic target in DOX-induced cardiomyopathy.

## 1. Introduction

Doxorubicin (DOX) is an effective chemotherapeutic drug but its clinical benefit is limited by its cardiotoxicity. Use of DOX results in progressive cardiotoxicity and leads to heart failure. DOX-induced cardiomyopathy is characterized by irreversible cardiac dysfunction and heart failure with poor prognosis [1]. Despite significant advances in cardio-oncology over the past decade, the molecular mechanisms that underlie DOX-induced cardiotoxicity have yet to be fully elucidated. The etiology of DOX-induced cardiotoxicity has been attributed to a multitude of mechanisms, which includes oxidative stress, mitochondrial dysfunction [2], metabolic disorders [3], dysregulated autophagy [4,5], ferroptosis [6], and apoptosis [7]. Various drugs are currently available for preventing DOX-induced cardiac injury, including enalapril, metformin, and dexrazoxane, which has displayed interesting cardioprotective effects in both in vitro and in vivo situations across many heart failure models [8,9,10]. The exact mechanism underlying the action of these drugs remains elusive, and their potential utilization in the management of DOX-induced cardiac toxicity is yet to be investigated. Mitochondria are essential for the maintenance of cellular ATP levels, which sustain the myocardium energy metabolism. Recent studies suggest that mitochondria are the key target for DOX-induced cardiomyopathy [11]. Our previous study reported that DOX interrupted oxidative phosphorylation and ATP production in H9c2 myoblasts, resulting in mitochondria impairment and cardiomyocytes apoptosis [12]. Disruption of the electron transport chain and inhibition of mitochondrial respiration in cardiomyocytes and reactive oxygen species (ROS) generation induced by mitochondrial dysfunction are thought to be critical for the pathogenesis of DOX-induced cardiomyopathy.

Cytochrome *c* oxidase (COX) is the terminal enzyme of the mitochondrial respiratory chain, which is a key site of cellular oxygen consumption and is essential for oxidative phosphorylation in the form of ATP. Mammalian COX is composed of 13 subunits, of which COX1, COX2, and COX3 are encoded by mitochondrial DNA and form the catalytic core of the enzyme, whereas the other 10 subunits are encoded by nuclear DNA and participate in the assembly and regulation activity of the enzyme [13]. COX deficiency mostly affects high metabolic tissues, such as the brain and muscle, manifesting in encephalopathy and cardiomyopathy [14,15,16]. To date, a majority of deleterious variants leading to COX deficiency has been reported in the nuclear-encoded assembly factors. Cytochrome *c* oxidase subunit 5A (COX5A), a nuclear-encoded subunit of COX, is located on the matrix side of COX and plays as an assembly factor. It has been reported that COX5A binds specifically with 3, 5-diiodothyronine, thus abrogating the allosteric ATP inhibition of COX [17]. Previous reports showed that down-regulation of COX5A significantly decreases COX activity, leading to mitochondrial dysfunction, pulmonary arterial hypertension, lactic academia, hypoglycemia, growth delay, and failure to thrive [18,19]. Furthermore, upregulation of COX5A significantly decreased the apoptosis in neurons and promoted neuronal outgrowth, thus protecting neurons from hypoxic ischemic injury [20]. In addition, a recent study proved that reduction in COX5A results in impairment of COX activity, which leads to heart failure [21]. However, the role of COX5A and its potential mechanism in DOX-induced cardiotoxicity remain largely unexplored.

In this study, we investigated the cardioprotective effects of overexpressing COX5A in cardiomyocytes and mice treated with DOX, and explored its possible underlying mechanisms. Thus, we identified COX5A as a potential therapeutic target for DOX-induced cardiomyopathy.

## 2. Results

### 2.1. COX5A Is Downregulated in the Heart Tissue of Patients with Dilated Cardiomyopathy

**Figure 1:**
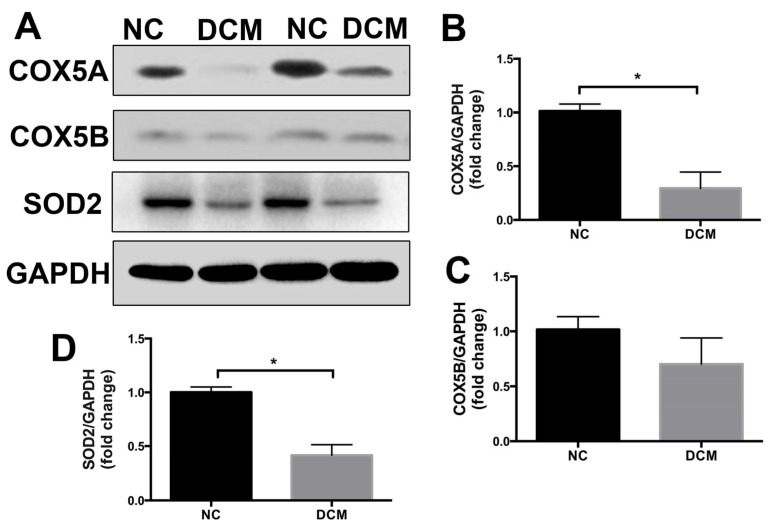
COX5A expression in the failing hearts. (**A**–**D**) Western blot analysis of COX5A, COX5B, and SOD2 expression in rejected healthy donor hearts and end-stage heart failure patients with DCM. Data are expressed as mean ± SEM. *n* = 4, * *p* < 0.05 vs. the normal control (NC) group.

We quantified COX5A, COX5B, and SOD2 protein levels in heart samples from patients with end-stage dilated cardiomyopathy (DCM) or subjects without obvious cardiovascular diseases, and observed that COX5A and SOD2 were clearly down-regulated in heart tissues with DCM (Figure 1). Although COX5B showed a trend of reduced expression in heart samples with DCM, there was no statistical significance between the two groups. These data suggest that downregulation of COX5A in cardiomyocytes may be involved in the development of heart failure in patients with DCM.

### 2.2. COX5A Is Decreased by DOX in the Heart of Mice and H9c2 Cardiomyoblasts

**Figure 2:**
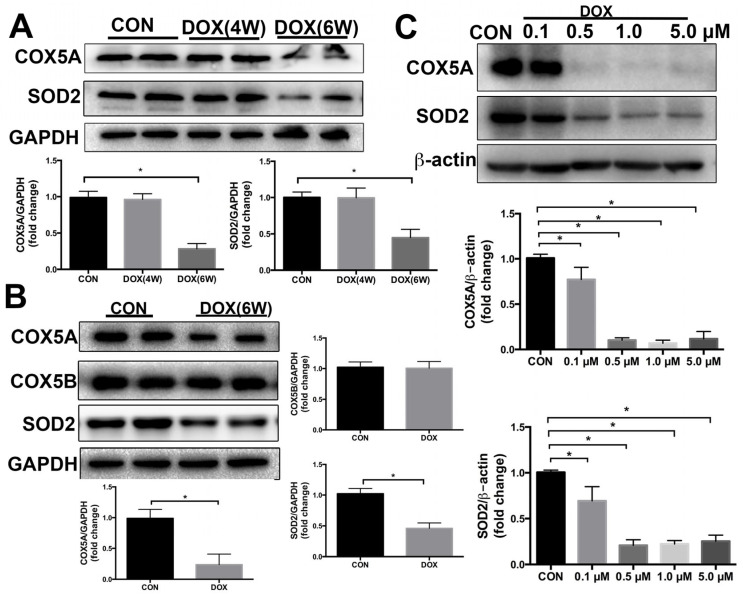
COX5A expression is decreased by DOX. (**A**,**B**) Western blot analysis of COX5A and SOD2 expression in mice heart tissues following DOX treatment for 4 weeks and 6 weeks, *n* = 6. (**C**) Western blot analysis of COX5A and SOD2 expression in H9c2 cells after treatment with DOX at various concentrations for 24 h, *n* = 6. Data are expressed as mean ± SEM. * *p* < 0.05 vs. the control (CON) group.

Mice were intraperitoneally injected DOX once weekly for 3 weeks with a cumulative dose of 18 mg/kg DOX. As shown in Figure 2A,B, 4 weeks after the first DOX treatment it had no significant effect on the protein expression of COX5A, but 6 weeks after the first DOX treatment it significantly diminished this expression in mice myocardium. In myocardium, changes in protein expression levels of mitochondrial antioxidants SOD2 roughly paralleled changes in COX5A expression levels at different time points of the DOX treatment. Additionally, we evaluated COX5B expression in the myocardium of mice after a 6-week treatment with DOX, and found no significant difference in COX5B expression compared to controls (Figure 2B).

Subsequently, H9c2 cardiomyoblasts were treated with DOX at different concentrations (0, 0.1, 0.5, 1.0, and 5.0 μM) for 24 h. In accordance with the in vivo results, 0.5 μM or higher DOX dramatically downregulated the protein expression of COX5A and SOD2 in H9c2 cells as compared with that in the control group (Figure 2C). Treatment with 0.5 μM DOX for 24 h did not significantly affect the protein expression of COX5B in H9c2 cells compared to the control group (Figure S1A).

COX5A was downregulated by DOX in vivo and in vitro, together with a concurrent decrease in SOD2 protein levels. These findings suggest a crucial role for COX5A in DOX-induced cardiomyopathy while also pointing to an association between COX5A and the expression of SOD2.

### 2.3. COX5A Upregulation Attenuates DOX-Induced Cardiotoxicity in Mice

**Figure 3:**
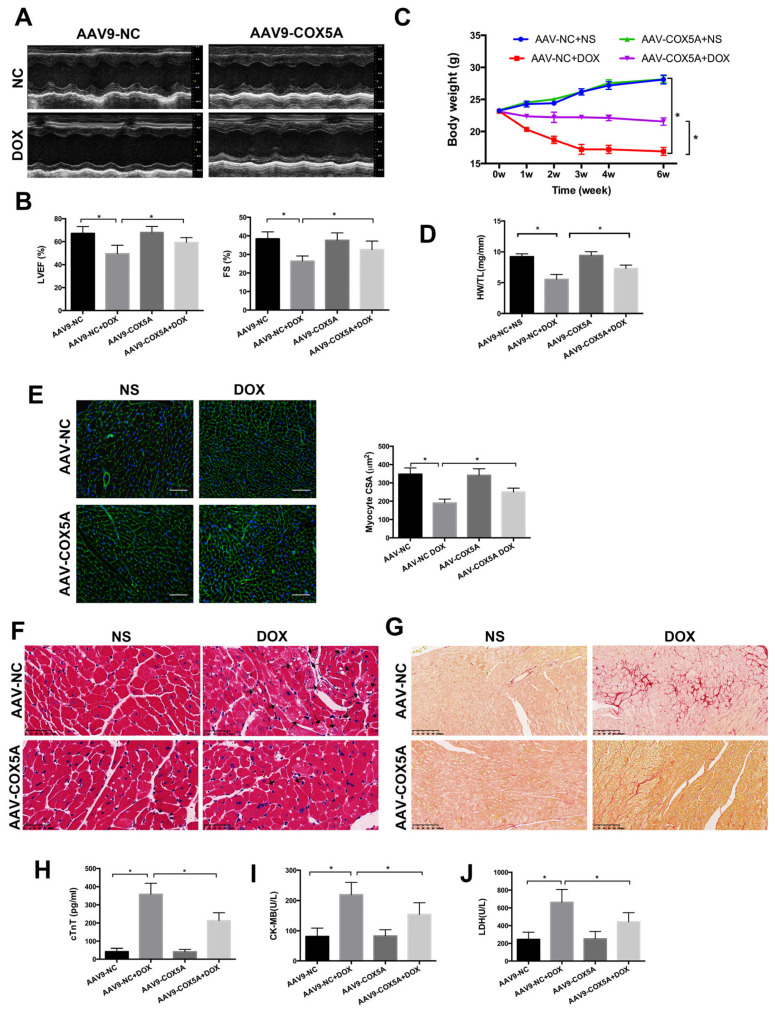
COX5A protects against DOX-induced cardiomyopathy in mice. (**A**) Representative M-mode echocardiograms for each group 6 weeks after DOX injection. (**B**) Echocardiographic quantification of LVEF and FS. (**C**) Body weight alterations with DOX treatment for different durations. (**D**) Statistical analysis of HW/TL. (**E**) Representative WGA-Alexa Fluor 488 conjugate stained heart sections and analysis of cardiomyocyte cross-sectional areas (CSA). Scale bar = 100 μm. (**F**) Representative histopathological findings of heart sections stained with H&E. Black arrows indicate extensive cytoplasmic vacuolization. Scale bar = 50 μm (**G**) Representative histopathological manifestations of heart sections stained with Sirius Red. Scale bar = 100 μm. (**H**) Analysis of serum level of cTnT by Elisa. (**I**,**J**) Biochemical determination of CK-MB and LDH serum levels. Data are expressed as mean ± SEM. * *p* < 0.05.

To explore the function of COX5A in DOX-induced cardiomyopathy, we specifically overexpressed COX5A in myocardium via a tail vein injection of recombinant adeno-associated virus serum type 9 carrying COX5A (AAV9-COX5A) and negative control (AAV9-NC) before DOX treatment. As shown in Figure 3A,B, COX5A overexpression improved the DOX-induced cardiac dysfunction, as indicated by the increased LVEF and FS. Furthermore, we found that cardiac-specific overexpression of COX5A attenuated DOX-induced body weight loss and heart weight loss, which raises the possibility of its clinical use (Figure 3C,D). COX5A overexpression alleviated DOX-induced myocardial injury, as indicated by alleviated heart atrophy, attenuated myocardial disarrangement, reduced cytoplasmic vacuolization and fibrosis, as well as decreased DOX-induced elevation of serum cTnT, CK-MB and LDH (Figure 3E–J). Therefore, we concluded that upregulation of COX5A attenuated DOX-induced cardiomyopathy in mice.

### 2.4. COX5A Overexpression Reduces DOX-Induced Oxidative Stress and Apoptosis in Mice

**Figure 4:**
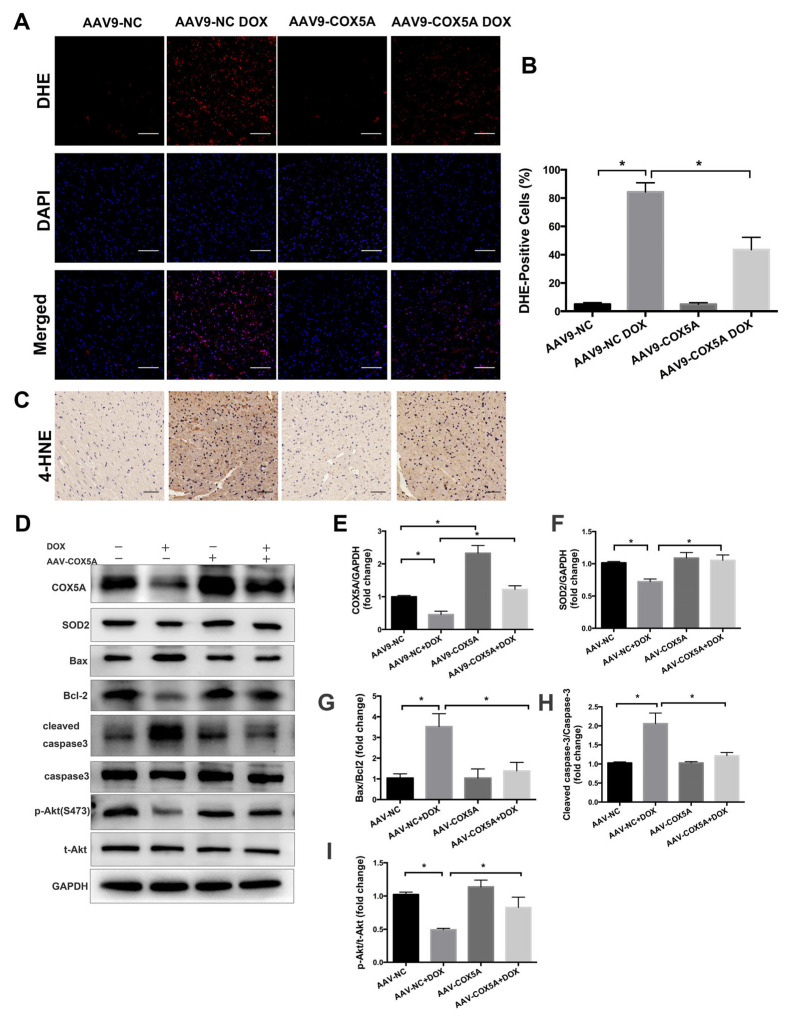
COX5A attenuates DOX-induced oxidative stress and apoptosis in mice. (**A**) Representative DHE staining images. Oxidized DHE intercalates into DNA and the nuclei appear in red. Scale bar = 100 μm. (**B**) Quantitative analysis of DHE-positive nuclei. (**C**) Representative IHC staining images of 4-HNE. (**D**–**I**) Western blot analysis of the protein level of COX5A, SOD2, Bax/Bcl-2, cleaved caspase-3/caspase-3, and p-Akt(S473)/t-Akt. Data are expressed as mean ± SEM. * *p* < 0.05.

Oxidative damage is suggested to be the key cause of DOX-induced cardiotoxicity; therefore, we used DHE staining and immunohistochemistry of 4-HNE to detect oxidative stress in myocardium. DOX treatment resulted in increased ROS production and lipid peroxidation in the myocardium, and COX5A overexpression markedly inhibited DOX-induced oxidative stress (Figure 4A–C). The myocardial protein level of COX5A was remarkably increased in mice following AAV9-COX5A transfection (Figure 4D,E). In addition, Western blot showed that COX5A overexpression significantly upregulated SOD2 expression in DOX-treated mice hearts (Figure 4D,F). Excessive ROS generation resulted in lipid peroxidation and increased induction of the mitochondrial permeability transition pore, which may trigger cardiomyocyte apoptosis and contribute to the progression of cardiac dysfunction; we subsequently assessed the role of COX5A in cardiomyocyte apoptosis. As shown in Figure 4D,G–I, overexpression of COX5A notably inhibited DOX-induced cardiomyocyte apoptosis as confirmed by Western blot results showing that upregulation of COX5A decreased the expression of Bax, cleaved caspase-3 and increased the Bcl-2 and p-Akt (Ser473) level.

### 2.5. COX5A Overexpression of Improved Mitochondrial Energy Metabolism in Mice Treated with DOX

**Figure 5:**
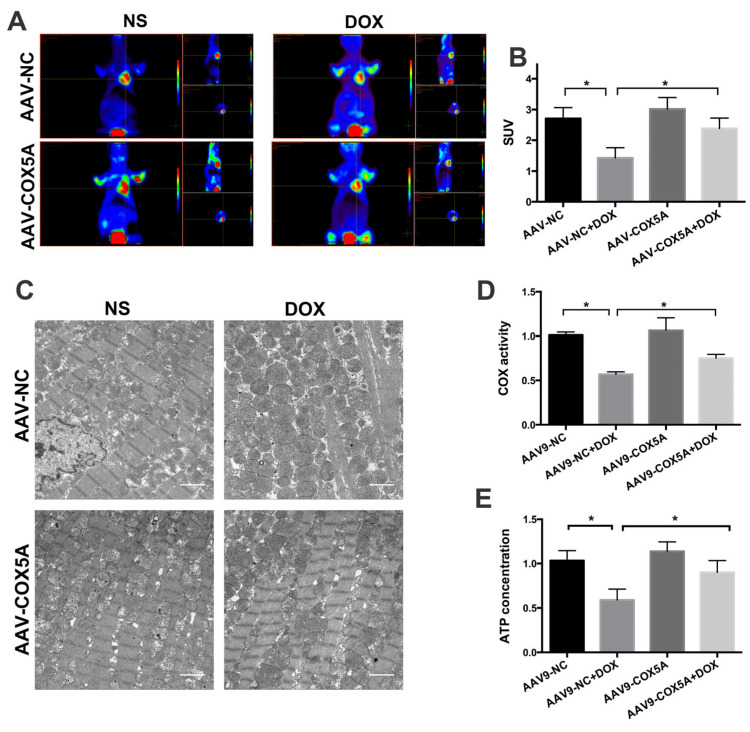
COX5A improves myocardial energy metabolism in mice. (**A**) Representative images of myocardial glucose uptake, determined by PET/MRI using 18F-FDG. (**B**) Quantitative analysis of SUV value. (**C**) The ultrastructure of mitochondria in the heart of mice. (**D**) Quantitative analysis of cytochrome *c* oxidase (COX) activity of mitochondria in the heart of mice. (**E**) Quantitative analysis of ATP production of myocardium of mice. Data are expressed as mean ± SEM. * *p* < 0.05.

To evaluate the metabolic status of mice hearts, we used positron emission tomography tomography/magnetic resonance imaging (PET/MRI) scanning employed fludeoxyglucose (18F-FDG) to assess glucose uptake. The standard uptake value (SUV) was adjusted with the body weights, radiation doses, residual radiation doses, and the duration between the injection and scan. As shown in Figure 5A,B, DOX treatment significantly decreased the SUV in mice hearts. Notably, compared to the AAV9-NC + DOX group, the mean SUV was significantly higher in the AAV9-COX5A + DOX group, suggesting that COX5A overexpression reserves DOX-induced viable myocardium loss and preserves energy metabolism. Subsequently, we observed the mitochondrial ultrastructure using transmission electron microscopy to assess the effect of COX5A on DOX-induced mitochondrial damage. Given that mitochondria disorganization may lead to reduced activity of mitochondrial respiratory enzymes and energy generation in hearts, we next evaluated the mitochondrial function by measuring COX (the terminal enzyme of the respiratory chain) activity and ATP levels. COX activity and ATP levels were significantly decreased in the mitochondria of DOX-treated mice compared with control mice and were significantly reserved by the overexpression of COX5A (Figure 5D,E).

### 2.6. COX5A Upregulation Alleviates DOX-Induced Oxidative Stress, Mitochondrial Dysfunction and Cardiomyocyte Apoptosis In Vitro

**Figure 6:**
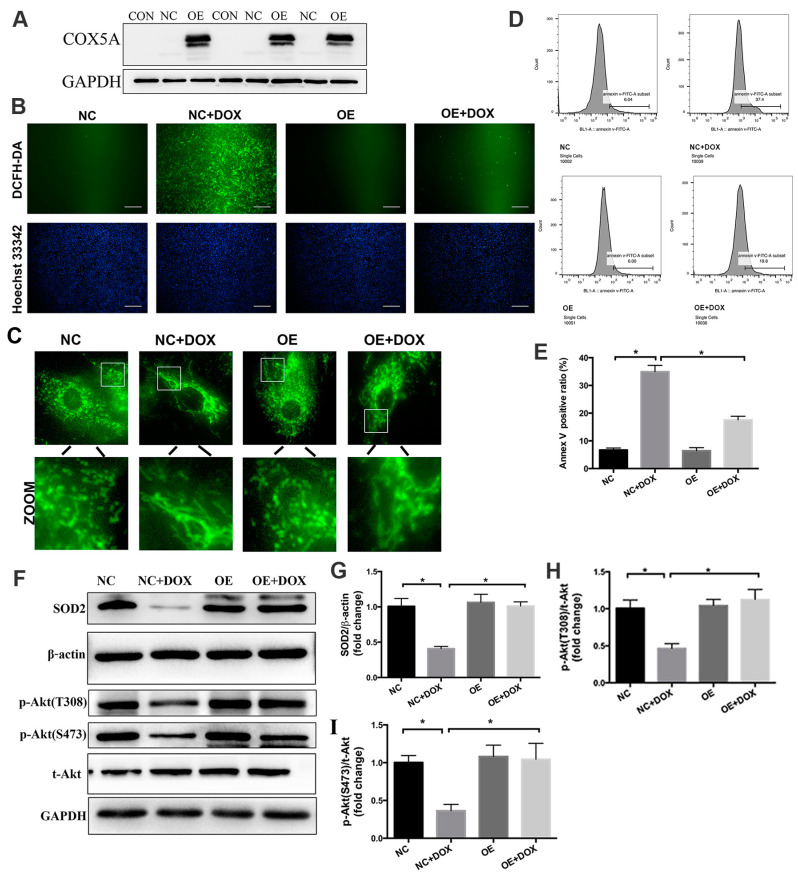
COX5A alleviates DOX-induced oxidative stress and cardiomyocyte apoptosis in vitro. (**A**) Expression of COX5A in H9c2 cells after transfection of lentivirus-COX5A and lentivirus-NC by Western blot. (**B**) DCFH-DA staining to detect ROS level. Nuclei were counterstained using Hoechst 33,342. Scale bars = 100 μm. (**C**) The morphology of mitochondria in H9c2 cells were stained with MitoTracker Green probe. (**D**) Annexin V-FITC staining followed by flow-cytometric analysis. (**E**) Quantitative analysis of Annexin V positive ratio. (**F**–**I**) Western blot analysis of the protein level of SOD2, p-Akt(T308)/t-Akt, and p-Akt(S473)/t-Akt. Data are expressed as mean ± SEM. * *p* < 0.05.

To further verify the protective role of COX5A in DOX-induced cardiomyocyte injury, H9c2 cardiomyoblasts were treated with COX5A-overexpressing lentivirus and a negative control lentivirus before DOX treatment. COX5A was successfully overexpressed in H9c2 cells following lentivirus transfection, and the transduced cell clones were then screened (Figure 6A). DCFH-DA staining and Western blot analysis confirmed that COX5A upregulation attenuated DOX-induced oxidative stress in cardiomyocytes as it attenuated ROS generation and increased the SOD2 protein level (Figure 6B,F,G).

Subsequently, we used MitoTracker Green FM to observe the mitochondrial structure of H9c2 cells. As shown in Figure 6C, mitochondria were disorganized, prolonged and accumulated around the nucleus with DOX treatment, and COX5A upregulation relieved DOX-induced mitochondrial disorganization.

Flow-cytometric analysis of FITC-Annexin V staining demonstrated that DOX treatment caused marked apoptosis in H9c2 cells, which was notably reversed by upregulation of COX5A (Figure 6D,E). In line with the results in vivo, we found that COX5A overexpression significantly attenuated DOX-induced the downregulation of p-Akt (Thr308) and p-Akt (Ser473) and exerted protective effects on oxidative stress and apoptosis in vitro (Figure 6F,H,I).

### 2.7. PI3K/Akt Signaling Is Responsible for the COX5A-Mediated Protective Role against DOX-Induced Cardiotoxicity

**Figure 7:**
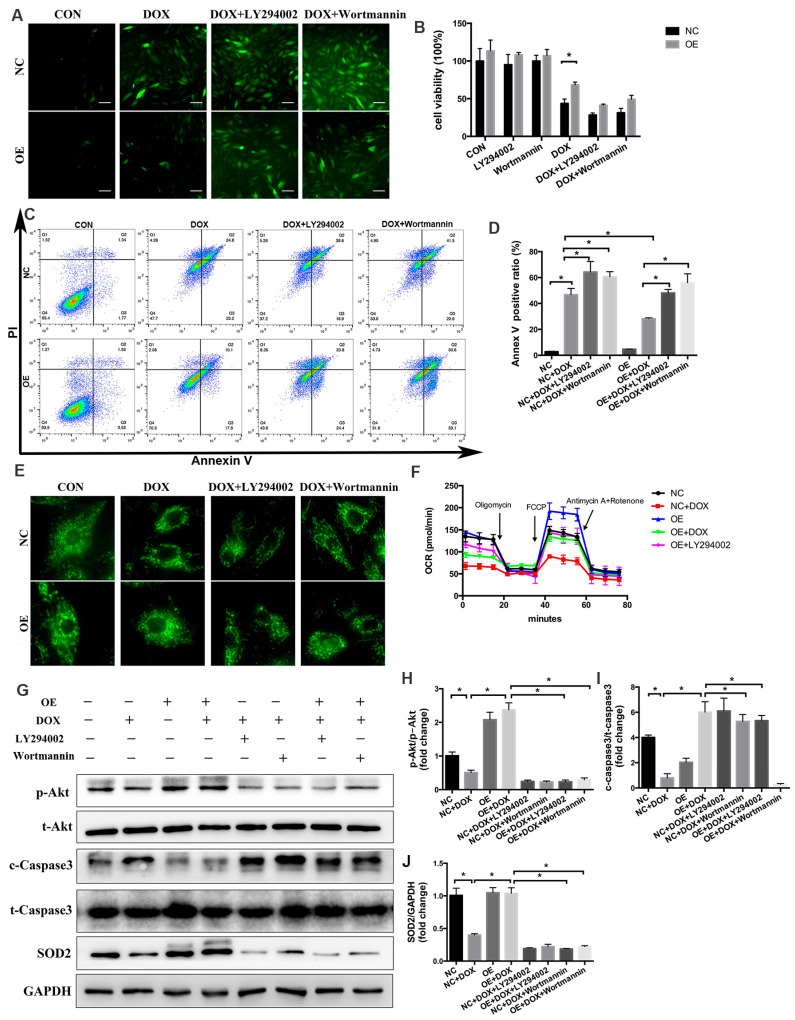
PI3K/Akt signaling is responsible for COX5A-mediated protective role in DOX-induced cardiotoxicity. (**A**) DCFH-DA staining to detect ROS level. Scale bars = 100 μm. (**B**) Cell viability detected by CCK-8 assay. *n* = 8. (**C**) Annexin V-FITC/PI staining followed by flow-cytometric analysis. (**D**) Quantitative analysis of of Annexin V positive ratio. *n* = 4. (**E**) The morphology of mitochondria in H9c2 cells stained with MitoTracker Green probe. (**F**) OCR measurements by means of the Seahorse^TM^ XFe96 extracellular flux analyzer at baseline and after addition of oligomycin, FCCP, and rotenone + antimycin A. (**G**–**J**) Western blot analysis of the protein level of p-Akt(S473)/t-Akt, cleaved caspase-3/t-caspase-3, and SOD2. Data are expressed as mean ± SEM. * *p* < 0.05.

As we observed, overexpression of COX5A both in vivo and in vitro mitigated DOX-induced inactivation of the PI3K/Akt pathway, which was further confirmed by the increased protein level of p-Akt (Figure 4D,I, and Figure 6F,H,I). To gain evidence that the protective roles of COX5A were mediated by PI3K/Akt activation, we treated H9c2 cells with either PI3K inhibitor LY294002 or Wortmannin. As shown in Figure 7A–F, PI3K inhibition almost completely abolished the beneficial role of COX5A in oxidative stress, cardiomyocyte apoptosis and mitochondrial dysfunction. LY294002 at the concentration of 10μM decreased the levels of ATP production, maximal respiration and spare capacity in OE cells, to a level similar to those in NC cells (Figure 7F). Furthermore, Western blot results showed that the protective roles of COX5A in DOX-induced oxidative stress and cardiomyocyte apoptosis diminished by PI3K/Akt inhibition decreased the expression of p-Akt (Ser473), SOD2 and increased cleaved caspase-3 level (Figure 7G–J). Collectively, our data showed that PI3K/Akt signaling was responsible for the COX5A-mediated protective role in oxidative stress, mitochondrial dysfunction and cardiomyocyte apoptosis.

## 3. Discussion

In this study, we demonstrated that COX5A alleviated cardiac dysfunction, improved the energy metabolism, reduced the susceptibility of cardiomyocytes to oxidative damage, and attenuated cardiomyocyte apoptosis in DOX-treated mice. Overexpressing COX5A via lentivirus transfection of H9c2 cells in vitro revealed similar results to those observed in vivo. Mechanistically, PI3K/Akt signaling was responsible for the COX5A-mediated protective role against DOX-treated H9c2 cells.

Increasing evidence suggests that mitochondrial dysfunction is significant in DOX-induced cardiomyopathy [11]. COX5A, as a nuclear-encoded subunit of COX, regulates mitochondrial respiration, based on its removal, modification or mutation leading to COX deficiency, resulting in mitochondrial diseases [21]. Yu et al. [16] found that cardiac ischemia-reperfusion (I/R) induced a 42% decrease in COX5A following prolonged I/R in the left ventricular myocardium. When they administered cardiac ischemic preconditioning before I/R, the COX5A was completely protected, which suggests an important regulation of COX. Our data demonstrated that, in the myocardium of DOX-treated mice, the COX5A protein level revealed no change 2 weeks after the final DOX treatment, but significantly decreased 4 weeks after the final DOX induction. In our in vitro experiment, the COX5A protein level decreased 24 h after 0.5 μM or a higher DOX stimulation.

DOX treatment destroyed myocardial redox homeostasis, resulting in vacuolar degeneration, mitochondrial dysfunction, and apoptosis of cardiomyocytes [1]. COX5A was involved in oxidative phosphorylation and is considered to be a key regulator of oxidative metabolism [21]. We previously reported that COX5A overexpression attenuated mitochondrial dysfunction and inhibited the apoptosis of DOX-treated H9c2 cells [12]. The cardioprotective roles of COX5A were further explored in this study in DOX-induced cardiomyopathy. In the study, we observed that the expression of COX5A was abundant in the myocardium, which was markedly reduced in response to DOX. Therefore, we forced COX5A overexpression in vivo and in vitro exposed to DOX.

Oxidative stress-mediated mitochondrial dysfunction is regarded as a major contributor to DOX-induced cardiotoxicity [22]. DOX treatment inhibits mitochondrial biogenesis, disrupts the mitochondrial structure and function, and prevents mitochondrial metabolism and self-repair, which eventually contributes to cardiomyocytes toxicity. A previous study showed that COX5A participates in COX assembly and involves ROS and redox-regulated steps, thus preventing oxidative stress [23]. SOD2 is a powerful ROS-scavenging enzyme in mitochondria [24]. A recent study has shown that patients with SOD2 defective may experience harmful increases in ROS in the neonatal heart, leading to the rapid development of heart failure and death [25]. Studies in mice have shown that the knockout of SOD2 causes the onset of DCM and subsequent death due to heart failure [26,27]. In recent studies, researchers have found that treatment with DOX resulted in a reduction in SOD2 protein levels in cardiomyocytes [28]. Interestingly, SOD2 overexpression attenuated doxorubicin-induced apoptosis and oxidative stress in cardiomyocytes, and improved survival and cardiac function in mice treated with DOX [29,30]. In agreement, our results also demonstrated that SOD2 significantly downregulated in the heart of DCM patients compared to that of healthy controls (Figure 1). Furthermore, we investigated the expression of the mitochondrial antioxidant SOD2 in the DOX-induced cardiomyopathy mice model and in H9c2 cells stimulated with DOX. In myocardium, changes in the protein level of the mitochondrial antioxidant SOD2 roughly paralleled changes in COX5A expression levels at different time points (Figure 2A,C). COX5A overexpression significantly decreased ROS generation in vivo and in vitro. Our findings are in line with those of previous studies showing that COX5A downregulation produces excessive ROS, which results in oxidative damage [31]. In our study, it was observed that COX5A was able to restore the protein level of SOD2 upon exposure to DOX. However, the silencing of SOD2 eliminated the favorable effect of COX5A on oxidative stress and cardiomyocyte apoptosis, as evidenced by increased ROS generation and the upregulation of cleaved caspase-3 (Figure S3). The protective roles of COX5A against DOX-induced cardiotoxicity may be partially mediated by regulating the expression of SOD2. DOX increases the ROS concentration in cardiomyocytes and causes apoptosis or necrosis. However, the presence of COX5A upregulated SOD2 and helped reduce ROS accumulation, thereby preventing apoptosis or necrosis.

4-hydroxy-2-nonenal (4-HNE), the main product of lipid peroxidation by ROS, can cause inhibition of COX activity [32]. COX is the rate limiting of oxidative phosphorylation in mitochondria. Mitochondria produce about 95% of ATP through oxidative phosphorylation, which plays a pivotal role in the myocardial energy metabolism. In addition to being a power source, mitochondria are the origin of apoptosis [33]. Mitochondria dysfunction results in impaired COX activity, a shift in the metabolic substrate utilization pattern, the disruption of ion homeostasis and excessive production of ROS [34]. Mitochondria injury is central to DOX-induced cardiac dysfunction and cell death [35]. Furthermore, the inhibition of ATP production could also induce apoptosis. Our results support the notion that the ATP level was decreased and apoptosis was induced following DOX treatment. COX5A overexpression attenuated mitochondrial structure impairment and increased 18F-FDG uptake, COX activity, and ATP levels. In addition, upregulation of COX5A attenuated the mitochondrial respiration dysfunction and energy metabolism disorder in DOX-treated H9c2 cells. In terms of OCR, the mitochondrial respiratory function was characterized by basal respiration, maximal respiration, and ATP production. Our data corroborate those reported by a previous study that COX5A abnormal expression is associated with impaired COX activity and decreased levels of cellular ATP [36]. Therefore, we concluded that overexpression of COX5A attenuated the disorder of the mitochondrial energy metabolism in DOX-induced cardiotoxicity.

In addition to being an energy source, mitochondria are the origins of the intrinsic pathway of apoptosis. DOX treatment results in the opening of mitochondrial pores, the decrease in MMP and the swelling of mitochondria, which lead to apoptosis [33]. In response to DOX treatment, pro-apoptotic Bcl-2 family proteins are transformed into oligomers, forming macropores in the outer membrane of mitochondria, resulting in the release of apoptotic factors including cytochrome *c* into the cytoplasm and initiating a cascade of apoptotic events that lead to cell death [37]. Here, we discovered that COX5A protected from myocardial loss in vivo and in vitro. In COX5A overexpressing mice, the cleaved caspase-3 protein level was significantly reduced. Caspase-3 is involved in myocardial apoptosis, and the inhibition of caspase-3 activation has a cardioprotective role in DOX-induced cardiotoxicity [22,38,39]. In contrast, Bcl-2 is an anti-apoptotic protein, which plays an important role in maintaining the mitochondrial structure and function and inhibiting apoptosis by interacting with Bax [40]. In our result, we showed that upregulation of COX5A could prevent DOX-caused mitochondria-mediated apoptosis, as evidenced by the decreasing Bax/Bcl-2 ratio and cleaved caspase-3. Our data are consistent with the fact that COX5A levels are correlated with cell survival [20].

PI3K/Akt signaling is an important regulator of cell death and muscle growth. A recent study showed that inhibition of PI3K and DOX treatment led to significant cardiac dysfunction accompanied by weight loss and heart atrophy [41]. Our study strongly supported the fact that DOX induced significant weight loss, reduced heart mass and heart atrophy in mice. A growing number of studies revealed that inactivation of Akt was responsible for DOX-induced cardiotoxicity, and enhanced Akt phosphorylation promoted the survival of cardiomyocytes and prevented DOX-induced cardiac dysfunction [42]. Consistent with these data, we found that overexpression of COX5A could activate Akt and protect cardiomyocytes from oxidative stress, mitochondrial dysfunction, and apoptosis, which could be almost completely reversed by PI3K/Akt inhibition. Our present findings provide the evidence that COX5A protected against DOX-induced cardiomyopathy via PI3K/Akt signaling.

In summary, we have provided evidence that COX5A upregulation in cardiomyocytes results in the attenuation of DOX-induced oxidative stress, mitochondrial dysfunction and cardiomyocyte apoptosis. Furthermore, PI3K/Akt signaling was responsible for a COX5A-mediated protective role in DOX-induced cardiotoxicity. Our study shows that COX5A is a promising therapeutic target against DOX-induced cardiotoxicity.

## 4. Materials and Methods

### 4.1. Myocardial Samples from Normal and Failing Human Hearts

Myocardial samples from patients with end-stage DCM post-heart transplantation were obtained from Zhongshan Hospital, Fudan University. Hearts used as controls with normal cardiac function and no history of cardiovascular disease were obtained from donors whose hearts were rejected for transplant. All patients or their family members gave their written, informed consent. For Western blot analysis, four end-stage failing human hearts and four rejected healthy donor hearts were studied. The study was conducted according to the guidelines of the Declaration of Helsinki and approved by the Ethics Committee of Zhongshan Hospital, Fudan University.

### 4.2. Animals and Experimental Protocols

Male wild-type C57BL/6J mice (8–9 weeks old) were purchased from Vital River Laboratory Animal Technology (Beijing, China). The mice were fed with standard chow and water and maintained in a temperature-controlled environment under a 12-h light/dark cycle. Mice were randomly divided into two groups (*n* = 10): the vehicle control group (CON) and the DOX treatment group (DOX). DOX (Sigma Aldrich, St. Louis, MO, USA) was dissolved in saline. Mice in the CON group received the following vehicles: equal volume intraperitoneal injection of normal saline (NS). Mice in the DOX group received DOX via an intraperitoneal injection (6 mg/kg body weight) once weekly for 3 weeks (cumulative dosage 18 mg/kg). To specifically overexpress COX5A in the myocardium, mice received a single intravenous injection of adeno-associated virus 9 (AAV9) carrying COX5A (AAV9-COX5A) or a negative control (AAV9-NC) via the tail vein at a concentration of 2× 10^11^ viral genome per mouse (male wild-type C57BL/6J mice, 6 weeks old). AAV9-COX5A and AAV9-NC were generated by the Hanbio Biotechnology Co. (Shanghai, China). Some 4 weeks after AAV9 injection, the mice were exposed to DOX via an intraperitoneal injection (6 mg/kg body weight) once weekly for 3 weeks (cumulative dosage 18 mg/kg) to generate DOX-induced cardiomyopathy or an equal volume of NS as a normal control. Mice were observed daily and weighed every week; 4 weeks after the final DOX treatment, cardiac functions were evaluated by echocardiography, and then were sacrificed with sodium pentobarbital (50 mg/kg; i.p.). Mice hearts together with the tibia were collected to calculate the heart weight/tibia length ratios (HW/TL). All animal experimental protocols and procedures were performed strictly according to the Laboratory Animal Guide of Fudan University and approved by the Experimental Animal Ethic Committee of Fudan University.

### 4.3. Echocardiography Evaluation

Transthoracic echocardiography was performed using a Vevo 770 high-resolution in vivo imaging system (Vevo770, Visual Sonics Inc., Toronto, ON, Canada) equipped with a RMV 707B scan head. Mice were anesthetized with isoflurane in oxygen. M-mode images were obtained when the heart rates of mice were stably maintained at 450–500 bpm. The left ventricular ejection fraction (LVEF) and left ventricular fractional shortening (LVFS) were measured. All records were averaged for 5 consecutive cardiac cycles.

### 4.4. Immunohistochemical Staining

Paraffin-embedded heart specimens were sliced to 5 μm, deparaffinized in xylene, and dehydrated in graded alcohol. Endogenous peroxidase activity was inhibited with 3% hydrogen peroxide and boiled in antigen retrieval buffer containing citratehydrochloric acid. Subsequently, the sections were blocked with 10% BSA for 2 h. After washing with PBS, sections were incubated with primary antibodies 4-HNE (Abcam, Cambridge, UK) overnight at 4 °C. HRP-polymer conjugate was added and incubated for 10 min. After washing with PBS, the DAB chromogen was added. Sections were counterstained with hematoxylin and examined with an Olympus BX-51 light microscope (Olympus, Tokyo, Japan).

### 4.5. Positron Emission Tomography (PET)

A PET scan was performed to acquire the standardized uptake value (SUV) via a MadicLab PET/MR system (Madic Technology Co., Shandong, China) to detect the survival myocardium and assess its viability. Briefly, the mice were weighed in advance and injected with 300 μCi of 18-FDG via the tail vein. After 30 min, the mice were scanned with PET. During the scanning, the mice were anesthetized with isoflurane to ensure no restlessness. Three-dimensional reconstruction was performed after PET scanning. The SUV of 18-FDG in the myocardial tissue was analyzed and calculated.

### 4.6. Transmission Electron Microscopy (TEM)

To demonstrate the ultrastructure of mitochondria, the heart tissue was cut into a 1 × 1 × 1 mm-sized patch and fixed with ice-cold 2.5% glutaraldehyde. After washing with PBS, all samples were fixed by 1% OsO4 and embedded in epoxy resin. Ultrathin sections were stained with uranyl acetate and lead citrate. Tecnai TEM (FEI, Hillsboro, OR, USA) was used for observation. All analyses were performed blind to the observer.

### 4.7. Tissue Mitochondria Isolation and COX Activity Assay

Heart tissue mitochondria were isolated using a tissue mitochondria isolation kit (Sigma Aldritch, St. Louis, MO, USA) according to the manufacturer’s protocol. The reagents (analysis buffer, enzyme dilution buffer, and reduced ferrocytochrome *c*) were prepared in advance according to the manufacturer’s protocol of the cytochrome *c* oxidase assay kit (Sigma Aldritch, St. Louis, MO, USA). A reaction system (volume to 1.05 mL) was prepared and the mixture was turned up and down: blank tube: 950 μL analytical buffer + 100 μL enzyme dilution buffer, sample tube: 950 μL analytical buffer + (100-x) μL enzyme dilution buffer + X μL mitochondrial suspension. At the beginning of the reaction, 50 μL of iron cytochrome *c* substrate solution was added and turned up and down to mix well. We immediately read the OD value of 550nm wavelength on the spectrophotometer. The COX activity of the sample was calculated.

### 4.8. Adenosine Triphosphate (ATP) Assay

The myocardial ATP content was measured using an ATP assay kit (Beyotime, Shanghai, China). In brief, heart tissues were harvested and analyzed according to the manufacturer’s instructions. The relative light unit was measured with a luminometer.

### 4.9. Hematoxylin and Eosin (H &E) Staining and Sirius Red Staining

The heart tissue was fixed in 4% paraformaldehyde followed by paraffin embedding and cut into 5 μm sections. Paraffin sections were deparaffinized and gradually rehydrated in xylene and serial dilutions of ethanol to 70% ethanol. Cardiac sections were stained with H&E and picrosirius red staining solution for histological examination.

### 4.10. Wheat Germ Agglutinin (WGA) Staining

To outline cardiomyocyte cell membranes for cross-sectional area measurement, cardiac sections were immersed in Fluor 488 conjugated wheat germ agglutinin (WGA) solution (Thermo Fisher Scientific, Waltham, MA, USA) for 10 min, and washed with PBS three times. The fluorescence images were obtained using a Leica fluorescence microscope (Leica, Wetzlar, Germany).

### 4.11. cTnT, LDH and CK-MB Level Assay

Serum was collected and the concentration of cardiac isoform of Tropnin T (cTnT) was measured using a mouse troponin T, cardiac muscle (TNNT2) ELISA kit (Cusabio, Wuhan, China). Lactate dehydrogenase (LDH) and creatine kinase isoenzymes (CK-MB) were measured using an automatic biochemical analyzer (Rayto, Shenzhen, China).

### 4.12. Dihydroethidium (DHE) Staining

To detect the tissue production of reactive oxygen species (ROS), fresh and frozen myocardium sections were incubated with 10 μM DHE (Sigma Aldritch, St. Louis, MO, USA) for 1 h in the dark. DAPI was counterstained to visualize the nuclei. The images were obtained using a Leica fluorescence microscope (Leica, Wetzlar, Germany). The numbers of DHE-positive nuclei and the total nuclei were counted from 50 random fields per group.

### 4.13. Cell Culture and Lentivirus Transfection

H9c2 cardiomyoblasts were obtained from the Cell Bank of the Type Culture Collection of the Chinese Academy of Sciences (Shanghai, China). Cells were cultured in DMEM (Thermo Fisher Scientific, Waltham, MA, USA) containing 10% of fetal bovine serum (Thermo Fisher Scientific, Waltham, MA, USA), and 100 U/mL penicillin-streptomycin (Hyclone, Logan, UT, USA) in a humidified atmosphere at 37 °C with 5% CO_2_. Lentiviral vectors were constructed by the Hanyin Co. (Shanghai, China), and were employed to overexpress COX5A in H9c2 cells. The COX5A-overexpressing lentivirus and a negative control lentivirus (NC-lentivirus; Hanyin Co., Shanghai, China) were prepared and titered to 10^8^ transfection units per milliliter. The transfected cell clones were designated as overexpression (OE) and negative control (NC) groups.

### 4.14. Flow-Cytometric Analysis

Cell apoptosis was analyzed using the FITC-Annexin V detection kit (BD Biosciences, San Jose, CA, USA). H9c2 cells were treated with 0.5 μM DOX (Selleck, Houston, TX, USA) for 24 h; harvested cells were then washed with cold flow cytometry binding buffer. After incubation with FITC-Annexin V and propidium iodide (PI), cell apoptosis was detected using a FACSCalibur (BD Biosciences, San Jose, CA, USA). Data were analyzed using Flowjo software (BD Biosciences, San Jose, CA, USA).

### 4.15. DCFH-DA Staining and CCK-8 Assay

Cell ROS production was evaluated by DCFH-DA staining (Beyotime, Shanghai, China). Briefly, H9c2 cells were stained with DCFH-DA (5.0 μmol/L) in the dark at 37 °C for 30 min and were then visualized in a blinded manner using an Olympus fluorescence microscope (Olympus, Tokyo, Japan). Cell viability was determined using the CCK-8 assay kit (Beyotime, Shanghai, China) according to the manufacturer’s protocol.

### 4.16. Mitochondrial Respiration Analysis

The NC and OE groups of H9c2 cells were plated at 9 × 10^3^ cells/well in Seahorse^TM^ XFe 96-well pre-coated microplates (Agilent Technologies, Santa Clara, CA, USA). The cells were treated with DOX (0.5 μM) for 24 h in a cell incubator. The mitochondrial inhibitors oligomycin (1.0 μM), carbonyl cyanide-p-trifluoromethoxyphenylhydrazone (FCCP; 1.0 μM), and rotenone + antimycin A (1.0 μM) were added to the cells sequentially. The OCR was measured using the Seahorse^TM^ XFe96 extracellular flux analyzer (Agilent Technologies, Santa Clara, CA, USA), calculating the basal respiration, maximal respiration, ATP production, and spare respiratory capacity, which were key parameters of the mitochondrial function.

### 4.17. Western Blot Analysis

Heart tissues were homogenized and H9c2 cells were lysed in RIPA buffer (Beyotime, Shanghai, China) containing protease and phosphatase inhibitors (Thermo Fisher Scientific, Waltham, MA, USA) and supernatants were collected after centrifugation. The protein concentration was determined using the BCA assay kit (Beyotime, Shanghai, China). Equivalent amounts of protein were loaded to the 10–12% sodium dodecyl sulfate polyacrylamide gel for electrophoresis. The proteins were then transferred to polyvinylidene fluoride membranes (Millipore, Bedford, MA, USA). The membranes were blocked in Tris-buffered saline + 0.1% of Tween-20 (TBST) containing 5% BSA and then incubated with primary antibodies against COX5A (Abcam, Cambridge, UK), SOD2 (Abcam, Cambridge, UK), Bax (Abcam, Cambridge, UK), Bcl-2 (Cell Signaling Technology, Beverly, MA, USA), cleaved caspase-3 (Cell Signaling Technology, Beverly, MA, USA), t-caspase-3 (Cell Signaling Technology, Beverly, MA, USA), p-Akt(Thr308) (Cell Signaling Technology, Beverly, MA, USA), p-Akt(Ser473) (Cell Signaling Technology, Beverly, MA, USA) and t-Akt (Cell Signaling Technology, Beverly, MA, USA) overnight at 4 °C. Subsequently, the membranes were incubated with a secondary antibody conjugated with horseradish peroxidase (Bioworld, Visalia, CA, USA) after three washes with TBST. Parallel immunoblots with antibodies against glyceraldehyde-3-phosphate dehydrogenase (GAPDH) and β-actin in TBST served as the loading control (Kangchen Biotechnology, Shanghai, China). Finally, the bands were detected using an enhanced chemiluminescence detection system (Millipore, Bedford, MA, USA) and quantified with Image Lab software (Bio-Rad, Hercules, CA, USA).

### 4.18. Statistical Analysis

Data were presented as the mean ± SEM. A Student’s *t*-test was conducted for the comparison of two groups, while the one-way ANOVA with a Bonferroni post hoc test was employed for the comparison of multiple groups [43]. Data with *p* < 0.05 were considered statistically significant. All data were analyzed by GraphPad Prism 6.0 software (GraphPad Prism Software, San Diego, CA, USA).

## Data Availability

The data and materials used to support the findings of this study are available from the corresponding author upon request.

## Supplementary Materials

The following supporting information can be downloaded at: https://www.mdpi.com/article/10.3390/ijms241210400/s1.
