# Supplementary material for: COX5A Alleviates Doxorubicin-Induced Cardiotoxicity by Suppressing Oxidative Stress, Mitochondrial Dysfunction and Cardiomyocyte Apoptosis
Source: Int J Mol Sci. 2023 Jun 20;24(12):10400. doi: 10.3390/ijms241210400 (PMC10299295; doi:10.3390/ijms241210400)
Supplement: Supplementary file 1 [file ijms-24-10400-s001.zip › ijms-2445860-supplementary.pdf]

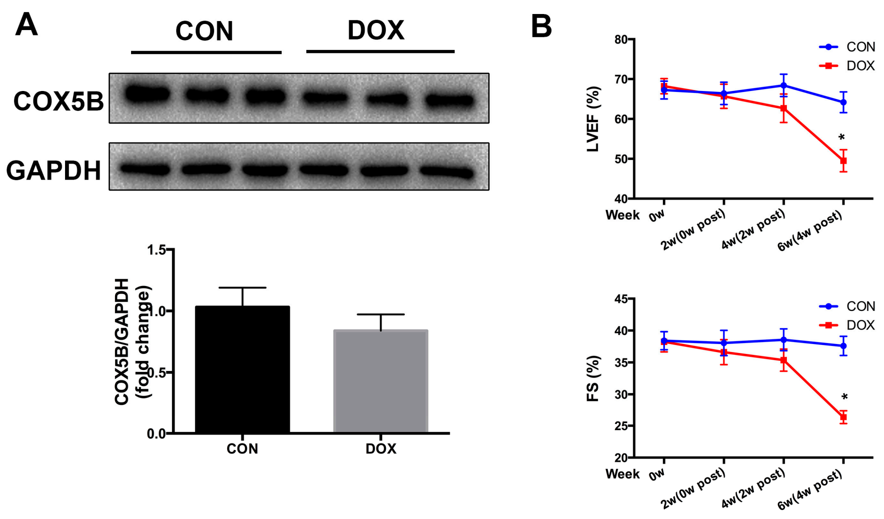

Figure S1. DOX-treatment on COX5B expression and cardiac function. **A.** Western blot analysis of COX5B expression in H9c2 cells treated with 0.5 μM DOX for 24 h. **B.** The changes of cardiac functional parameters (LVEF and FS) at different time post DOX treatment. Data are expressed as mean ± SEM. **P* < 0.05 vs. the normal control (CON) group.

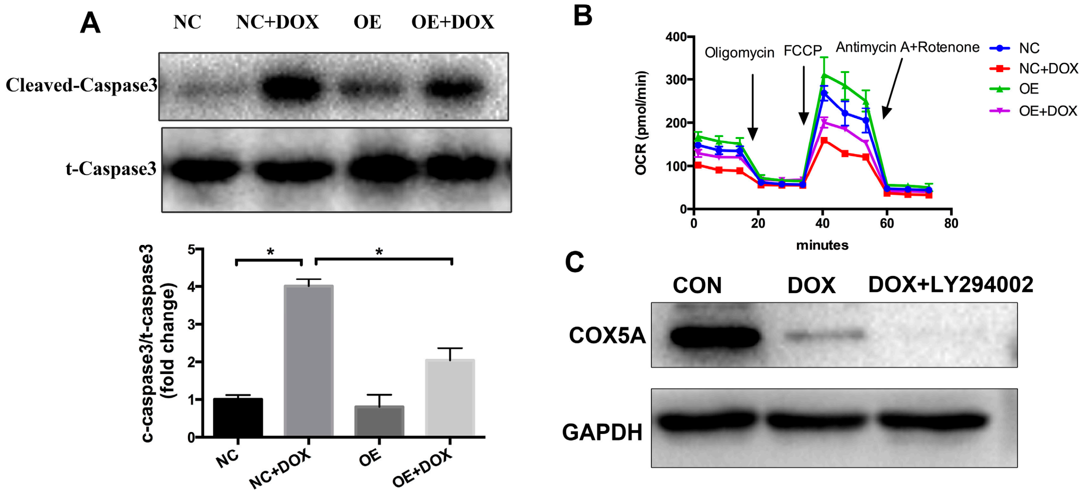

Figure S2. COX5A alleviates DOX-induced cardiomyocyte apoptosis and mitochondrial dysfunction *in vitro*. **A**. Western blot analysis of the protein level of cleaved casapase-3/t-caspase 3. **B.** OCR measurements by means of the Seahorse™ XFe96 extracellular flux analyzer at baseline and after addition of oligomycin, FCCP, and rotenone+antimycin A. **C.** LY294002 (10 μM) was used to pre-treat H9c2 cells for 2 hours, followed by continued treatment with 0.5 μM DOX for 24 hours. The expression of COX5A was detected using Western blot analysis. The results showed that treatment of DOX led to a significant decrease in the expression of COX5A, whereas combination treatment with DOX+LY294002 resulted in an even more substantial reduction of COX5A compared to DOX group alone. Data are expressed as mean ± SEM. **P* < 0.05.

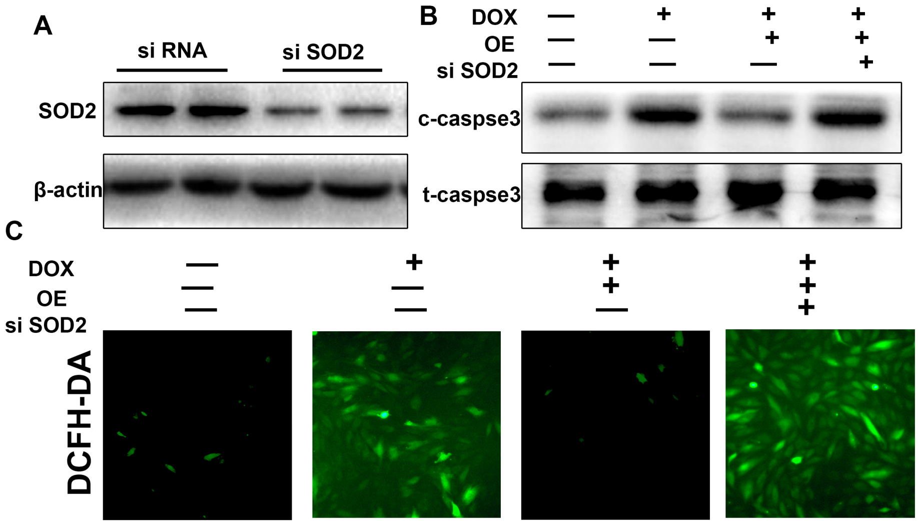

Figure S3. COX5A alleviates DOX-induced cardiomyocyte apoptosis and oxidative stress via upregulation of SOD2. **A**. Western blot analysis of the protein level of SOD2. **B.** Western blot analysis of the protein level of cleaved caspase-3 and t-caspase-3. **C.** DCFH-DA staining to detect ROS level.
